# Seven days of mixed‐method heat acclimation improved markers of cardiovascular and fluid‐regulatory strain during exercise‐heat stress

**DOI:** 10.1113/EP092681

**Published:** 2025-08-27

**Authors:** Daniel Snape, Barney Wainwright, Iain T. Parsons, Michael J. Stacey, David R. Woods, John O'Hara

**Affiliations:** ^1^ Carnegie School of Sport Leeds Beckett University Leeds UK; ^2^ Research and Clinical Innovation Royal Centre for Defence Medicine Birmingham UK; ^3^ School of Cardiovascular Medicine and Sciences King's College London London UK

**Keywords:** acclimation, adaptation, cardiovascular physiology, fluid‐regulatory, performance, thermal

## Abstract

A mixed‐method heat acclimation (HA) protocol may optimise performance by supporting the training taper while promoting thermal adaptation; however, the impact on cardiovascular and fluid‐regulatory adjustments to protect health is unknown. Therefore, we examined the effects of a mixed‐method heat protocol on physiological responses, including cardiovascular and fluid‐regulatory strain with exercise‐heat stress, and self‐paced performance in the heat. Twenty (15 males, five females) triathletes were randomised to 8 days of HA (HOT), or exercise in thermoneutral conditions (TEMP). A heat stress test (HST) comprising 45 min of cycling in a climatic chamber (32°C, 70% relative humidity) was performed on days 1, 5 (HOT only) and 8. Before and after the intervention, a cycling time trial was conducted in the same climatic conditions (days 0 and 10). Venous blood samples were analysed at rest and post‐HST (days 1 and 8 only) for the catecholamine product normetanephrine and the vasopressin surrogate copeptin. Following 7 days of HA (days 1 vs. 8) resting rectal temperature was significantly lower in the HOT compared to the TEMP group (−0.32 ± 0.36°C, *P =* 0.002). Normetanephrine was 24.3% lower after 7 days of HA (*P =* 0.012), and copeptin was 53.4% lower at the post‐HST time point (HOT vs. TEMP, *P =* 0.012). However, HA had no effect (0.3%, *P =* 0.984) on self‐paced performance in the heat. Mixed‐method HA elicited a progressive reduction in cardiovascular strain and a net reduction in fluid‐regulatory strain without improving self‐paced performance in the heat.

## Abstract (highlights)

- **What is the central question of the study?**
  Does 7 days of mixed‐method heat acclimation reduce thermal strain and markers of sympathetic activity and fluid regulation at rest and during exercise‐heat stress?
- **What is the main finding and its importance?**
  Seven days of mixed‐method heat acclimation resulted in a progressive reduction in cardiovascular strain, a net reduction in fluid‐regulatory strain, plasma volume expansion and a lower core body temperature in hot conditions. These findings add to the limited evidence regarding the effects of heat acclimation on reducing sympathetic activity and improving fluid regulation, factors likely to contribute to protecting kidney function.

## 1 INTRODUCTION

Heat acclimation (HA) is a highly valued method of preparation in athletes and support teams for the purpose of protecting athlete health and maximising performance in the heat. Core features of heat adaptation include plasma volume expansion, improved fluid regulation, decreased resting and exercising core and skin temperatures, and a reduction in exercising heart rate (Tyler & Notley, 2024). Whilst there has been a growing body of evidence on the best method to optimise the heat adapted phenotype (Tyler et al., 2024), these guidelines are primarily based on research in non‐elite populations, using fixed‐intensity protocols over consecutive days. Elite triathletes, however, undertake high training volumes and intensive training schedules due to the demands of preparing for racing in three different disciplines (Mujik, 2014). The introduction of sustained HA protocols represents an additive stressor in this athlete population, which independently can provide a further immune challenge (Hailes et al., 2011; Mitchell et al., 2002), and could increase the risk of ‘over‐reaching’ and ‘overtraining’ (James et al., 2024). Consequently, there is a practical need to develop HA strategies that stimulate adaptation while minimising health risks and avoiding disruption to training quality – an area where research is still developing (Casadio et al., 2017).

Alternative approaches to traditional exercise‐in‐heat protocols, such as post‐exercise hot water immersion (Zurawlew et al., 2018), training wearing thermal clothing (Lundby et al., 2021) and post‐training sauna use (Stanley et al., 2015), have been explored as more flexible tools for integrating HA into high‐performance training schedules. Exercise in a climatic chamber, however, allows athletes to practice with representative pacing and thermal/exertional perception (James et al., 2017), which may be critical for sport‐specific adaptation and competition performance (Daanen et al., 2018; Gibson et al., 2020). Mixed‐method (active and passive) HA protocols have been shown to reduce thermal strain and improve performance in hot conditions for various athletic populations, including match officials, endurance athletes and elite team sport players (Fenemor et al., 2022; James et al., 2024; Ruddock et al., 2016; Stephenson et al., 2019). However, despite these promising results, there is a clear lack of randomised controlled trials assessing the short‐term effectiveness of this approach, particularly in endurance‐trained individuals, and especially in relation to the biomarkers and physiological markers that could offer a more comprehensive understanding of heat adaptation status.

To maximise adaptation, it is widely understood that HA protocols must progressively increase the thermal impulse as the body becomes more adapted to heat stress (Tyler et al., 2024). One approach, known as controlled hyperthermia, involves manipulating core body temperature through environmental heat, clothing or exercise intensity to maintain a certain threshold (e.g. 38.5°C) via active or passive exposure. Although this method is challenging to implement in field settings, it overcomes limitations with practical approaches of HA prescription based on HR or self‐selected pace (Taylor et al., 2020). A combined approach – integrating controlled hyperthermia with post‐exercise hot water immersion – may therefore offer triathletes a feasible strategy for inducing meaningful heat adaptation while preserving training quality.

In addition to traditional markers of heat adaptation, such as core body temperature and heart rate, further insight may be gained by assessing endocrine and stress biomarkers. During exercise in the heat the autonomic nervous system (ANS) buffers increased competition for blood flow between cutaneous circulation and the skeletal muscle (Rowell, 1990). Observable measures of ANS disturbance and sympathetic activity include catecholamines and their nephrine metabolites (Bracken et al., 2009; Stacey, Delves et al., 2018). Plasma nephrines have been highlighted as potential markers of acclimatisation status, with reductions linked to decreased acute kidney injury (AKI) risk and improved regional perfusion following heat adaptation (Omassoli et al., 2019; Stacey, Delves et al., 2018). Similarly, copeptin, a stable and practical surrogate for arginine vasopressin (AVP), has emerged as a valuable biomarker for evaluating fluid‐regulatory strain and heat stress adaptation (Katan & Christ‐Crain, 2010). Lower copeptin concentrations after HA have been associated with reduced AKI and hyponatraemia risk (Omassoli et al., 2019). Including these biomarkers alongside traditional physiological measures could enhance the evaluation of heat adaptation status and allow for a more personalised and health‐protective approach to athlete preparation. Further research is required to examine plasma copeptin and normetanephrine concentrations in response to short‐term mixed‐method HA protocols, particularly in endurance athletes who may already possess partial HA from regular training. Exploring these responses will help practitioners better tailor HA programmes to optimise both performance and athlete well‐being in hot environments.

The aims of this study were to investigate the effects of a mixed‐method heat protocol – combining controlled hyperthermia sessions in a climatic chamber with post‐exercise hot water immersion – on physiological responses, including cardiovascular and fluid‐regulatory strain during exercise‐heat stress, and self‐paced performance in the heat. Our hypothesis was that a mixed‐method HA protocol would confer significant heat adaptation responses after 4 days of HA, with more complete adaptation after 7 days. We further hypothesised that 7 days of mixed‐method HA would significantly reduce thermal strain and improve self‐paced performance in the heat compared to a control group.

## 2 METHODS

### 2.1 Ethics statement and participant population

The study protocol gained institutional ethical approval (Reference: 84895, Leeds Beckett University) and was conducted in line with the principles expressed in the *Declaration of Helsinki*, 2013. Twenty (15 males, five females) triathletes provided written informed consent. All athletes completed a minimum of ≥5 h aerobic exercise per week and according to classifications by Pauw et al. (2013) met the criteria for performance levels 2 or 3, with none in level 1 (Table 1). Athletes had regularly competed in triathlons from sprint to ironman distance for >3 years. Female participants were eumenorrhoeic or using various forms of hormonal contraceptives.

**TABLE 1:** Baseline physical characteristics of the heat acclimation (HOT) and temperate exercise intervention (TEMP).

	HOT (*n* = 10, 7 male and 3 female)	TEMP (*n* = 10, 8 male and 2 female)	*P*
Age (years)	28 ± 6	31 ± 9	0.429
Body mass (kg)	70.5 ±9.4	76.1 ± 15.1	0.332
Height (cm)	176 ±9	179 ± 7	0.314
Body fat (%)	16.3 ± 5.5	18.4 ±5.9	0.441
${\dot{V}}_{O_{2}\max}$ (L min^−1^)	3.93 ± 0.74	4.20 ±0.89	0.478
${\dot{V}}_{O_{2}\max}$ (mL kg^−1^ min^−1^)	55.9 ± 9.7	56.1 ± 11.3	0.973
MRMP (W s)	325 ± 72	327 ± 71	0.960
HR_max_ (bpm)	182 ± 12	183 ± 12	0.914
LT (W)	188 ± 60	193 ± 54	0.848
LTP (W)	240 ± 66	248 ± 57	0.789

*Note*: Values represent the mean ± SD. The reported values of maximal oxygen consumption (${\dot{V}}_{O_{2}\max}$), maximum ramp minute power (MRMP), lactate threshold (LT) and lactate turnpoint (LTP) were measured under temperate conditions (20°C, 45% relative humidity).

### 2.2 Design of study

Participants were randomly assigned to independent groups, mixed‐method HA (HOT) or temperature exercise intervention (TEMP) using a random number generator software package (GraphPad Prism, version 8.1.0; GraphPad Software, San Diego, CA, USA). Environmental conditions (32°C, 70% relative humidity [RH]) during controlled hyperthermia sessions were specifically chosen to mimic the conditions athletes were anticipated to face during the Tokyo 2020 Olympic Games (Gerrett et al., 2019). All acclimation and performance trials were scheduled at the same time of day to limit the confounding effect of circadian rhythm variation (Waterhouse et al., 2005). Participants were also instructed to arrive in a euhydrated state, as demonstrated by a urine osmolality <700 mOsmol kg^−1^ and urine specific gravity <1.020 (Kenefick & Sawka, 2007). Participants were instructed to not complete any additional strength/endurance training throughout the duration of the study. The primary outcomes of this study were changes in physiological markers of heat adaptation (core temperature, heart rate, plasma volume change) and exercise performance during self‐paced cycling in the heat. Secondary outcomes, specified a priori, included changes in fluid‐regulatory and sympathetic biomarkers (copeptin and normetanephrine) to explore their potential role as practical indicators of heat adaptation status. The inclusion of these biomarkers was based on emerging evidence of their sensitivity to thermal strain and their relevance to health monitoring in both athletic and occupational heat stress contexts.

### 2.3 Preliminary testing

Participants’ stature (Seca, 220, Hamburg, Germany) and body mass (Seca, 770) were measured initially, and participants’ body fat (%) was assessed by dual‐energy X‐ray absorptiometry (GE Healthcare Lunar, Chicago, IL, USA). A graded exercise test (GXT) was performed on a calibrated cycle ergometer (Wattbike, Wattbike Atom X, Nottingham, UK). Participants initially cycled at 150 W and increased by 25 W every 4 min until a blood lactate (BLa) concentration >4.0 mmol L^−1^. BLa was measured from capillary blood and analysed using a biochemistry analyser (EKF, Biosen C‐Line, Cardiff, UK), calibrated following the manufacturer's instructions. This allowed for the determination of lactate threshold (LT), the intensity at which the BLa concentration exceeded the resting value, and lactate turn point (LTP), the intensity above which a distinct, sudden and sustained increase in BLa concentration occurred. Following a 15‐min recovery, participants cycled at 150 W and increased the power by 20 W min^−1^ until volitional exhaustion. This test allowed the identification of maximum heart rate (HR_max_), maximum ramp minute power (MRMP) and maximal oxygen uptake (${\dot{V}}_{O_{2}\max}$). Heart rate (HR) was recorded continuously during all exercise tests using telemetry data from a strap affixed to the chest of the participant (V800, Polar, Kempele, Finland). Measurements of expired air were taken using a calibrated breath‐by‐breath online gas analysis system (Cortex, Metalyzer 3B, Leipzig, Germany) and ${\dot{V}}_{O_{2}\max}$ was determined according to standard criteria of Winter et al. (2006).

### 2.4 20‐km cycling time‐trial

**FIGURE 1:**
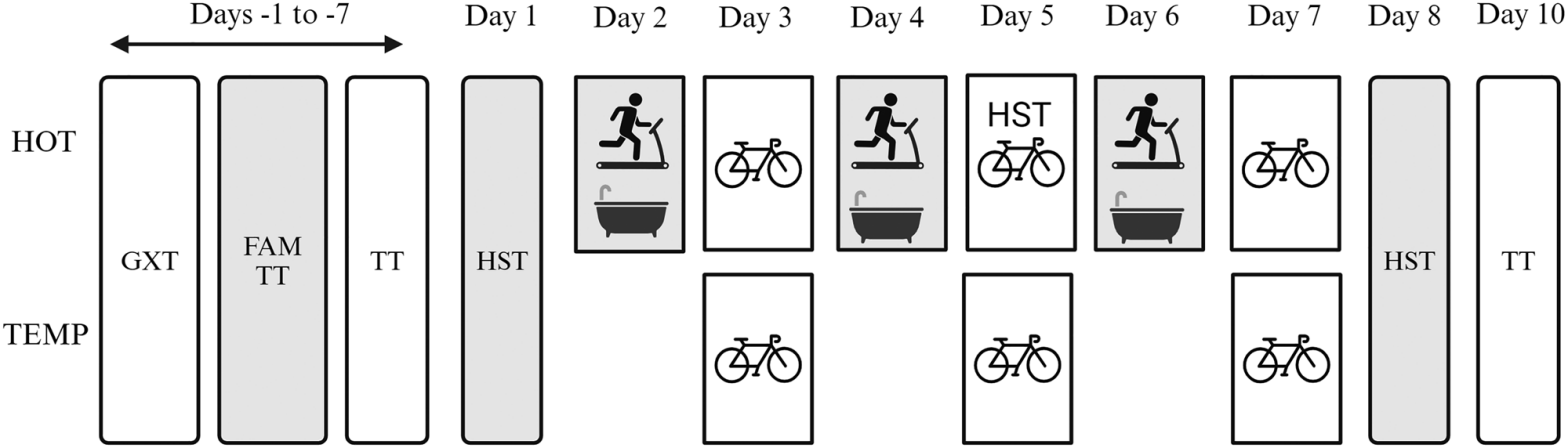
Schematic representation of the study protocol. FAM, familiarisation time‐trial; GXT, graded exercise test; HOT, heat acclimation group; HST, heat stress test; TEMP, temperate exercise group; TT, 20 km cycling time‐trial in the heat. Created with BioRender.com.

All participants completed an initial familiarisation 20‐km cycling time trial (TT) in temperate climate laboratory conditions (20.4 ± 1.2°C, 47 ± 11% RH), along with pre‐ and post‐intervention TTs (days 0 and 10) in hot, humid conditions (TT1: 31.9 ± 0.3°C, 73 ± 3% RH; TT2: 31.9 ± 0.4°C, 72 ± 6% RH) (see Figure 1). Each TT was performed on a Cyclus 2 ergometer (RBM elektronik‐automation GmbH, Leipzig, Germany). During trials in the heat, participants self‐inserted a rectal thermistor probe (Variohm, 4492, 400 series, Northamptonshire, UK) connected to a data logger (Grant, SQ2020 2F8, Royston, UK) approximately 10 cm proximal of the anal sphincter for the measurement of rectal temperature (*T*_re_). Towel‐dried nude body mass (NBM) was measured before and after the trials and sweat rate (L h^−1^) was estimated using changes in NBM from pre‐ to post‐exercise periods. Fluid intake and urine output was accounted for but not insensible respiratory water and metabolic losses, as these were considered negligible (Kenefick & Sawka, 2007). A 10‐min warm‐up for all TTs was standardised, involving 5 min of cycling at the participant's LT, followed immediately by 5 min of passive recovery. No verbal encouragement, feedback or music was permitted throughout the TT. Participants were instructed to complete the distance in the shortest possible time and were blinded to all measures except distance covered at 5‐km intervals. An electric fan was placed in front of the participants to provide convective cooling throughout (approx. 8.5 m s^−1^). HR was recorded continually and noted at 5‐km intervals along with *T*_re_, BLa, thermal sensation (TSS, Toner et al., 1986) and rating of perceived exertion (RPE; Borg, 1982).

### 2.5 Heat stress test

Heat stress tests (HSTs) were completed on days 1, 5 (HOT only) and 8 of the intervention within an environmental chamber (Sporting Edge, Basingstoke, UK) set to hot, humid conditions (32.0 ± 0.3°C, 71 ± 4% RH). Consequently, we report the effects of seven HA sessions (i.e. ‘short‐term’ HA) in the HOT group, although participants completed an eighth HA session. The HST involved 45 min of fixed‐intensity exercise on a cycle ergometer (Wattbike, Atom X, Nottingham, UK), adapted from Snape et al. (2023). Participants with a LT > 2.5 W kg^−1^ cycled at 2.5 W kg^−1^ during identical HSTs (*n* = 10), whereas those with a LT < 2.5 W kg^−1^ cycled at 2.0 W kg^−1^ (*n* = 10). Four skin temperature (*T*_skin_) loggers (Maxim Integrated Products, iButton, DS1922L, California (CA), USA) were affixed to the participant using Opsite Flexifix tape (Bunzl, Smith & Nephew plc, UK) and mean *T*_skin_ was calculated using standard equations of Ramanathan (1964). Sweat patches (3M, Tagaderm +Pad, Minnesota, USA) were applied to the forearm for the collection of localised sweat. Participants then entered the environmental chamber and rested in a seated position for 5 min to obtain baseline physiological and perceptual data. During exercise HR was monitored continuously, *T*_re_ at 2.5‐min intervals, and RPE, TSS and thermal comfort (TC) (Zhang et al., 2004) recorded at 5‐min intervals. Sweat rate was estimated from changes in NBM pre–post the HST, corrected for urine output and fluid intake. Fluid intake was restricted to water, consumed ad libitum, and placed in the environmental chamber for ∼20 min before the trial to equilibrate at room temperature. Venous blood samples were drawn at rest (PRE) prior to entering the environmental chamber and immediately post‐HST (POST) from an antecubital vein. Blood was analysed for the concentration of normetanephrine (NMET), copeptin, haemoglobin (Hb) and haematocrit (Hct).

### 2.6 Mixed‐method heat acclimation intervention (HOT)

Controlled hyperthermia sessions were completed on days 1, 3, 5, 7 and 8 of HA (Figure 1). During controlled hyperthermia sessions HR was measured throughout, with perceptual measures (RPE, TSS, TC) at 10‐min intervals over the 90‐min session. Fluid intake was monitored, with bottle weight measured pre‐ to post‐session to the nearest 0.1 g using weighing scales (Ohaus, C series, New Jersey, USA). Controlled hyperthermia exposures on days 3 and 7 involved participants cycling for 10 min at their LT, followed by a 20‐min period of intervals of 1‐min duration (1:1 min work: rest) alternating between the participants’ power output (PO) at LTP and LT. The aim of this session was to raise rectal temperature to 38.5°C within 30 min and maintain it between 38.5°C and 39.3°C for 45–60 min. Contrastingly, on days 1, 5 and 8 the first 45 min involved the cycling HST followed by a further 45 min of self‐adjusted cycling exercise via external PO to maintain *T*_re_ ≥38.5°C. Post‐exercise hot water immersion (HWI) sessions were performed on days 2, 4 and 6 of HA, structured to align with a triathlete's typical weekly running frequency when tapering for competition (Mujika, 2011, 2014). Participants ingested a gastrointestinal pill (BodyCap, e‐Celsius, Normandy, France) ≥6 h prior to their visit for the continuous measurement of gastrointestinal temperature (*T*_gi_). Participants initially completed a 30‐min treadmill run at a moderate intensity (RPE = 13) under temperate laboratory conditions (20.9 ± 0.2°C, 58 ± 2% RH). Approximately 4 min following the cessation of running, participants (clothed in swim shorts or a swimsuit) were submerged to the neck in the hot water bath (CET CryoSpas, CryoSpa Sport, Bangor, Northern Ireland) at a water temperature of 39.5 ± 0.1°C for up to 40 min. HR was recorded at 5‐min intervals using a pulse oximeter (Nellcor, PM10N, MN, USA), with perceptual measures of TSS and TC recorded at 5‐min intervals. Daily accumulated thermal load area under the curve (AUC), which was pre‐session core body temperature baseline corrected, was calculated for both controlled hyperthermia and post‐exercise HWI sessions.

### 2.7 Temperate climate exercise intervention (TEMP)

The TEMP group completed three 90‐min thermoneutral exercise sessions (20°C, 40% RH) on days 3, 5 and 7, along with 45‐min HSTs on days 1 and 8 only. In addition, the three thermoneutral exercise sessions included a 10‐min warm‐up on a cycle ergometer at the participants’ PO at LT, before completing high‐intensity intervals over a 20‐min period, followed by up to 60‐min steady state cycling at a PO equivalent to their LT, as described for controlled hyperthermia exposures. HR was measured throughout, with perceptual measures (RPE, TSS, TC) at 10‐min intervals over the 90‐min session. Sweat loss was measured from pre to post changes in NBM and accounted for fluid intake and urine output. These sessions were designed to match the cycling load from the controlled hyperthermia days in the HOT group.

#### 2.7.1 Analytical methods

Whole blood collected into a EDTA collection tube (32.332 Sarstedt, Akteingesellscaft & Co., Numbrecht, Germany) was immediately analysed for Hb and Hct using a haematology analyser (Horiba, Pentra, ES 60, Montpellier, France) calibrated according to manufacturer's instructions. Changes in plasma volume (PV) (∆PV) were estimated following the equation described by Dill & Costill (1974), from measured Hct and Hb. Sweat patches were analysed for Na^+^ concentration using a flame photometer (Jenway, PFP7 Flame Photometer, IL, USA). For plasma NMET another EDTA collection tube was centrifuged within 10 min of being collected, with serum collected into serum separator tube (Becton Dickinson, SSTTM II Advance, Franklin Lakes, NJ, USA) and allowed to clot for 30 min at room temperature before being centrifuged for serum copeptin. All samples were centrifuged for 10 min at 2683 *g*. Aliquots of plasma and serum were stored at −80°C until subsequent analysis. Plasma NMET was analysed via a liquid chromatography/tandem mass spectrometry method (coefficient of variation (CV): 4–12%). Serum copeptin was assayed using a benchtop analyser (Brahms, CT‐proAVP Kryptor Compact Plus, MA, USA) and had a CV of 2–10%. Hormone concentrations presented are corrected for changes in plasma volume.

### 2.8 Statistical analysis

Data were analysed using GraphPad Prism (Version 8.1.0). All data are reported as means ± standard deviation (SD) and statistical significance was set at *P <* 0.05. Results were assessed for normality using the Shapiro–Wilk test (*P* ≥ 0.05). The effects of time (days 1 vs. 8) and group (HOT vs. TEMP) on continuous variables were determined using a two‐way ANOVA for repeated measures. Where missing values presented a repeated measure design, a mixed‐effects model was used. If a significant interaction was observed, *post hoc* tests were completed using the Bonferroni correction for multiple comparisons (α = 0.05). The means of the HA group over HSTs (days 1, 5, 8) for physiological measures were analysed using a one‐way repeated measures ANOVA (parametric) or Friedman's test (non‐parametric) with Dunn's correction for perceptual measures (TSS and TC). Differences in participants’ baseline physical characteristics were assessed via an independent sample Student's *t*‐test. Mean changes in urinary specific gravity (USG), urine osmolality (UOSM), haematological measures and sweat sodium concentration during HSTs and pre‐trial USG, fluid intake and sweat rate during the 20‐km cycling TTs were evaluated using a paired sample *t*‐test (parametric).

## 3 RESULTS

Twenty‐two participants were assessed for eligibility and randomised to either the HOT (*n* = 12) or the TEMP (*n* = 10) group. Two participants were lost from the HOT group due to illness (*n* = 1) and personal reasons (*n* = 1) and were excluded from the analysis, resulting in 20 participants who completed all study visits.

### 3.1 Heat stress test: Physiological adaptations

**FIGURE 2:**
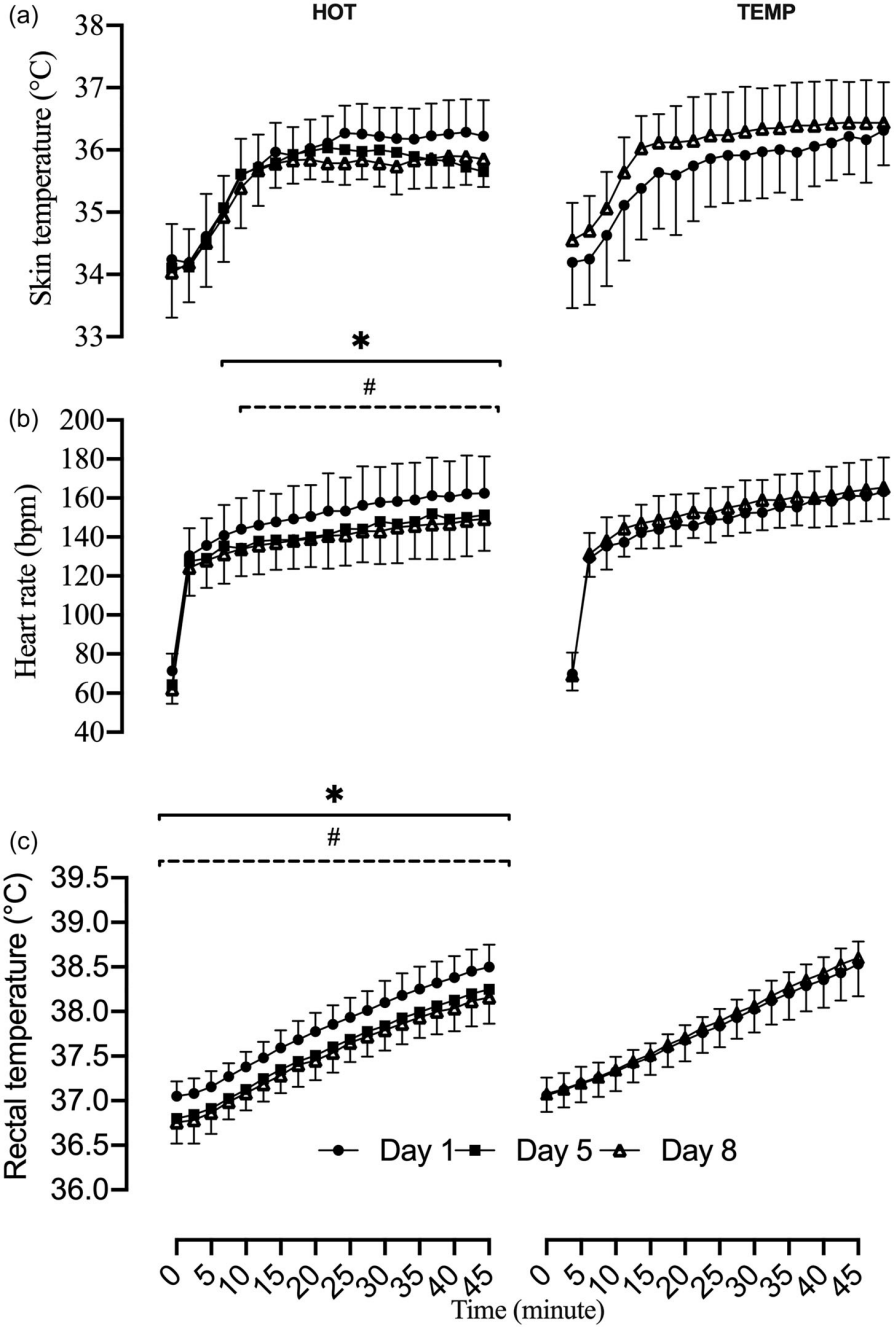
Changes in skin temperature (a), heart rate (b) and rectal temperature (c) during heat stress tests at days 1, 5 (HOT only) and 8 in the HOT and TEMP group. Main effect of time (*P <* 0.001), but no effect of intervention (*P =* 0.068) or interaction (*P =* 0.116) for skin temperature. Main effect of time, intervention and interaction (all: *P <* 0.001) for heart rate and rectal temperature. *Significant difference from days 1 to 8. ⧣Significant difference from days 1 to 5 in the HOT group.

A summary of resting, mean and peak physiological measures throughout each intervention are presented in Table 2. Following 7 days of heat acclimation (days 1 vs. 8), the change in resting *T*_re_ was greater in HOT compared to TEMP (HOT: −0.29 ± 0.26°C; TEMP: 0.02 ± 0.19°C; *P =* 0.002). The changes in mean *T*_re_ (HOT: −0.32 ± 0.19°C; TEMP: 0.04 ± 0.21°C; *P <* 0.001), mean *T*_skin_ (HOT: −0.34 ± 0.23°C; TEMP: 0.34 ± 0.60°C; *P =* 0.032) and peak *T*_re_ (HOT: −0.32 ± 0.19°C; TEMP: 0.07 ± 0.42°C; *P =* 0.010) after HA were also greater in HOT compared to TEMP. Mean *T*_re_ was lower at all time points (*P <* 0.001) after 4 (range: 0.23°C–0.27°C) and 7 days of HA (range: 0.23°C–0.35°C; Figure 2c). After 4 days of HA mean HR was lower from 10 to 45 min (*P* ≤ 0.023), and from 7.5 to 45 min (*P* ≤ 0.017) after 7 days of HA (Figure 2b). In contrast, no significant changes in the TEMP group were present at any time point for *T*_re_, HR or *T*_skin_ (*P* > 0.999).

**TABLE 2:** Physiological responses to cycling heat stress tests on days 1, 5 and 8 of heat acclimation (HOT) and days 1 and 8 of the temperate exercise intervention (TEMP).

	HOT			TEMP	
	Day 1	Day 5	Day 8	Day 1	Day 8
Resting					
*T*_re_ (°C)	37.05 ± 0.17	36.80 ± 0.21	36.76 ± 0.24*	37.06 ± 0.20	37.08 ± 0.21
*T*_skin_ (°C)	34.2 ± 0.6	34.1 ± 0.5	34.0 ± 0.7	34.2 ± 0.7	34.6 ± 0.6
HR (bpm)	71 ± 9	64 ± 10	62 ± 8	70 ± 11	70 ± 8
Mean					
*T*_re_ (°C)	37.87 ± 0.18	37.62 ± 0.15	37.55± 0.19*	37.82 ± 0.18	37.61± 0.43
*T*_skin_ (°C)	35.9 ± 0.4	35.7 ± 0.4	35.6 ± 0.4*	35.8 ± 0.6	36.1 ± 0.6
HR (bpm)	151 ± 16	141 ± 14	140 ± 16	150 ± 15	154 ± 14
Peak					
*T*_re_ (°C)	38.50 ± 0.25	38.25 ± 0.24	38.16 ± 0.29*	38.54 ± 0.25	38.61 ± 0.43
*T*_skin_ (°C)	36.5 ± 0.4	36.2 ± 0.4	36.1 ± 0.3*	36.5 ± 0.5	36.6 ± 0.6
HR (bpm)	165 ± 18	153 ± 15	152 ± 18*	166 ± 16	168 ± 15

*Note*: Values represent the mean ± SD. *Significant differences between groups at day 8 (*P <* 0.05). A significant interaction (*P* = 0.007) main effect of time (*P = *0.017) and group (*P = *0.046) was observed for resting *T*_re_. A significant interaction (*P <* 0.001) and main effect of time (*P = *0.006) for mean *T*_re_ and an interaction effect (*P =* 0.014) for peak *T*_re_. Resting *T*_re_ was lower at day 5 (*P = *0.040) and day 8 (*P = *0.018) in the HOT group compared to day 1, along with mean *T*_re_ (*P <* 0.001, *P* = 0.001, respectively) and peak *T*_re_ (*P* = 0.002, *P = *0.003, respectively). There was no interaction for resting HR (*P = *0.091), which was lower in the HOT group after seven days of HA (day 1 vs. day 8) (*P <* 0.001). Mean and peak HR observed a significant interaction (*P <* 0.001, *P = *0.003, respectively) and main effect of time (*P =* 0.023, *P =* 0.012, respectively). Mean and peak HR were lower at day 5 (*P = *0.012, *P = *0.004, respectively) and day 8 (*P = *0.022, *P = *0.011, respectively) in the HOT group compared to day 1. A significant interaction was present for mean (*P = *0.006) and peak (*P = *0.028) *T*_skin_. Mean and peak *T*_skin_ were lower at day 8 versus day 1 in the HOT group (*P* = 0.042, *P = *0.022, respectively), as well as day 1 versus day 5 for peak *T*_skin_ (*P = *0.018). HR, heart rate; *T*_re_, rectal temperature; *T*_skin_, skin temperature.

#### 3.1.1 Haematological, biochemical and sweat rate responses

**FIGURE 3:**
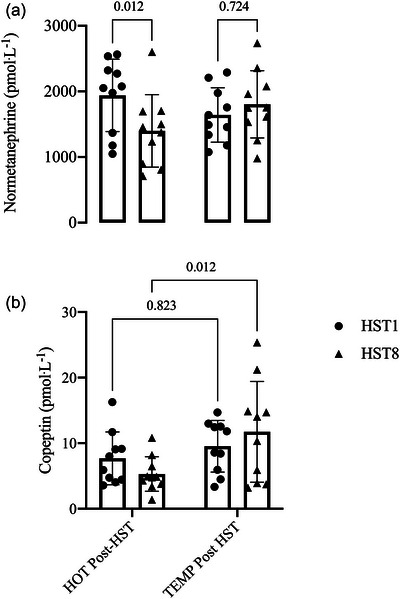
Mean (SD) and individual data points for normetanephrine (a) and copeptin (b) concentrations in the heat acclimation (HOT) and temperate climate exercise group (TEMP). Main effect of intervention (*P <* 0.001), no effect of time (*P =* 0.814) and no interaction (*P =* 0.070) for copeptin. Main effect of intervention (*P <* 0.001) and interaction (*P =* 0.037), but no effect of time (*P =* 0.485) for NMET.

Seven days of HA demonstrated a significant increase in resting PV compared to no change in the TEMP group (HOT: +9.6 ± 7.3%; TEMP: −0.3 ± 11.1%, *P =* 0.004). Sweat rate increased after 7 (+0.38 ± 0.33 L h^−1^; *P =* 0.015), but not 4 days of HA (+0.07 ± 0.41 L h^−1^; *P* > 0.999). There was no effect of 7 days of HA on sweat sodium concentration (*P =* 0.124), fluid intake (*P* > 0.999) pre‐trial USG (*P =* 0.451), UOSM (*P =* 0.481) and body mass (*P *> 0.999). For the TEMP group there were no significant differences in resting PV (−0.3 ± 11.1%; *P* > 0.99), body mass (*P =* 0.062), sweat rate (*P =* 0.577), sweat sodium concentration (*P =* 0.809) and fluid intake (*P* > 0.999) from pre‐ to post‐intervention. NMET concentration was reduced by −24.3 ± 7.7% after 7 days of HA (*P =* 0.012, Figure 3a). When comparing the HOT versus the TEMP group, copeptin concentration was significantly reduced at the post‐HST time point (−53.4, ± 64.8%, *P =* 0.012, Figure 3b).

### 3.2 Heat stress test: Perceptual adaptations

There was a significant interaction and main effect of time for peak RPE (*P =* 0.025 and *P =* 0.016) and peak TC (*P =* 0.011 and *P =* 0.002), along with a main effect of time in mean TC (*P =* 0.002) after 7 days of HA. In contrast, there was no change in the TEMP group pre‐ to post‐intervention (*P* > 0.05). Analysis revealed significant reductions in peak RPE (*P* = 0.012) and peak TC (*P =* 0.005) after 4 days of HA, with no change from days 5 to 8 in all perceptual measures (*P* > 0.05).

### 3.3 Heat acclimation and temperate climate exercise intervention protocols

Daily physiological responses to controlled hyperthermia days are displayed in Table 3. The time above the target *T*_re_ was significantly lower on day 5 compared to day 2 and 3 (−29 min, *P =* 0.043 and −22 min, *P =* 0.038, respectively). One‐way ANOVA showed no differences in accumulated thermal load within controlled hyperthermia (*P =* 0.095) or HWI days (*P =* 0.056). When comparing controlled hyperthermia sessions to temperature exercise days there were no differences in mean HR (*P =* 0.607) or power output (*P =* 0.257), suggesting cardiovascular strain and relative external load were matched between groups. The physiological and perceptual responses to post‐exercise HWI are displayed in Table 4. There were significant differences between HWI sessions for mean HR (*P <* 0.001); specifically mean HR was significantly lower during passive 1 compared with passive 3 (−7 bpm, *P =* 0.050). Furthermore, mean TC were significantly different between HWI sessions (*P =* 0.001), whereby TC was lower during passive 2 compared to passive 3 (−1 AU, *P =* 0.042); see Table 4.

**TABLE 3:** Physiological responses to heat stress test (HST) and controlled hyperthermia days in the heat acclimation (HOT) group.

	HST/Day 1	Day 3	HST/Day 5	Day 6	HST/Day 8
Time to 38.5°C *T*_re_ (min)	55 ± 18	34 ± 14	47 ± 14	49 ± 18	58 ± 22
Time above 38.5°C *T*_re_ (min)	30 ± 14	46 ± 20	39 ± 13	38 ± 18	18 ± 13
AUC (°C min^−1^)	102 ± 18	118 ± 23	117 ± 30	118 ± 19	104 ± 26
Sweat rate (L h ^−1^)	1.27 ± 0.50	1.69 ± 0.66	1.49 ± 0.51	1.44 ± 40	1.75 ± 0.53
HR (bpm)	151 ± 13	149 ± 10	145 ± 10	145 ± 11	141 ± 11
PO (W)	143 ± 28	155 ± 32	154 ± 27	161 ± 32	159 ± 34
PO at LT (%)	83 ± 28	88 ± 24	88 ± 26	91 ± 22	90 ± 25
PO at MRMP (%)	45 ± 10	49 ± 9	49 ± 9	50 ± 7	50 ± 9
Exercise duration (min)	82 ± 5	83 ± 6	81 ± 6	77 ± 13	77 ± 5

*Note*: Values represent the mean ± SD. The time above 38.5°C was significantly higher on day 3 (*P =* 0.043) and day 5 (*P* = 0.038) compared to day 8. Sweat rate was significantly higher (*P =* 0.032) and HR significantly lower (*P* = 0.019) on day 8 compared to day 1. AUC, accumulated thermal load area under the curve; HR, heart rate; LT, lactate threshold; MRMP, max ramp minute power; PO, power output; *T*_re_, rectal temperature.

**TABLE 4:** Physiological and perceptual responses to post‐exercise hot water immersion (HWI) on day 2, 4 and 7 of heat acclimation.

	Day 2	Day 4	Day 7
Moderate intensity treadmill run (RPE = 13)			
Mean HR (bpm)	155 ± 13	152 ± 12	150 ± 12
Mean *T*_gi_ (°C)	37.81 ± 0.25	37.73 ± 0.32	37.73 ± 0.34
Δ *T*_gi_ (°C)	1.36 ± 0.31	1.31 ± 0.43	1.37 ± 0.63
Post‐exercise HWI (water temperature = 39.5°C)			
Mean HR (bpm)	107 ± 6	104 ± 7	100 ± 11
Mean *T*_gi_ (°C)	38.57 ± 0.29	38.60 ± 0.23	38.55 ± 0.28
Δ *T*_gi_ (°C)	0.61 ± 0.48	0.79 ± 0.60	0.83 ± 0.50
Mean TSS (AU)	6.2 ± 0.5	6.1 ± 0.5	5.8 ± 0.6
Peak TSS (AU)	7.0 ± 0.6	6.6 ± 0.6	6.5 ± 1.0
Mean TC (AU)	3 ± 1	3 ± 1	2 ± 1
Immersion time (min)	35 ± 4	36 ± 4	39 ± 2
Participants completing 40‐min immersion (*n*)	3 (30%)	4 (40%)	7 (70%)

*Note*: Values represent the mean ± SD. Data for *n* = 10. Mean HR was significantly lower on day 7 compared to day 2 (*P =* 0.050). Mean TC was significantly lower on day 7 compared to day 4 (*P =* 0.042). AU, arbitrary units; HR, heart rate; RPE, rating of perceived exertion; TC, thermal comfort; *T*_gi_, gastrointestinal temperature; TSS, thermal sensation.

### 3.4 20‐km cycling TT in the heat

**FIGURE 4:**
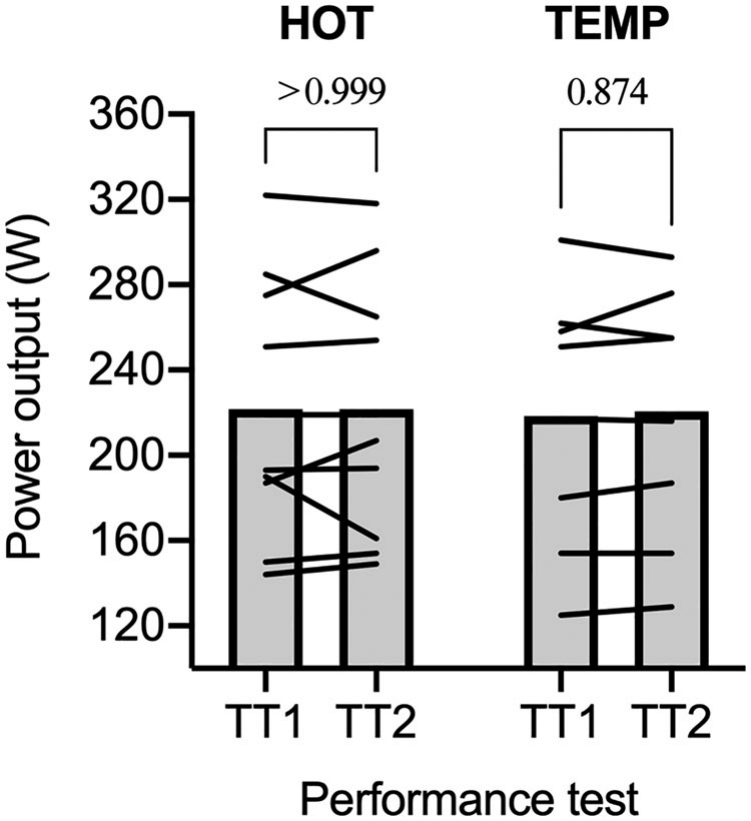
Performance trial (20 km cycling time‐trial [TT] in 32°C, 70% relative humidity) average power output at baseline (TT1) and post‐intervention (TT2) for the heat acclimation group (HOT, *n* = 10) and temperate exercise group (TEMP, *n* = 8). Bars are group mean; lines are individual participants. No main effect of time (*P =* 0.719), intervention (*P =* 0.942) or interaction (*P =* 0.744).

Two participants in the TEMP group did not complete the full 20‐km distance due to reaching *T*_re_ ≥ 39.7°C and so were removed from analysis. The average change in PO for the HOT group was 0.3%, indicating no significant difference after HA (*P =* 0.984; Figure 4). Analysis revealed a significant interaction and main effect of time for mean TSS (*P =* 0.035 and *P =* 0.001), whereby TSS was lower (−0.6 AU, *P <* 0.001) in the HOT group only. In addition, there was a lower mean RPE (−1 AU, *P =* 0.006), and end‐exercise *T*_re_ (−0.45°C, *P <* 0.001). No interaction (*P =* 0.587), main effect of time (*P =* 0.077) or group (*P =* 0.149) were observed in mean BLa.

## 4 DISCUSSION

A primary finding of the current study was that 4 days of mixed‐method HA was effective at reducing thermal strain in a hot, high‐humidity environment, with no further increase in resting and exercise HR, *T*_re_, RPE and TC with an additional 3 days. Our results provide novel evidence of diminished NMET and copeptin following a cycling HST after 7 days of mixed‐method HA. Reduced sympathetic activity and fluid regulatory stress, along with plasma volume expansion and a reduced core temperature, may be protective of kidney function in moderately trained individuals during exercise in the heat. Interestingly, no improvement in 20‐km cycling TT performance in the heat was evident following 8 days of HA, despite reductions in end‐exercise *T*_re_, mean TSS and mean RPE for the same absolute PO compared to the pre‐intervention TT.

The mixed‐method HA protocol was successful at lowering resting *T*_re_ (−0.25°C) and HR (−7 bpm) after four exposures, with an additional three exposures (7 days) showing no further resting adaptations in *T*_re_ (−0.04°C) or HR (−2 bpm). These responses are in accordance with previous literature that has reported that these adaptations happen at a rapid rate (Nielsen et al., 1993) and are not further improved after a longer exposure (Moss et al., 2020). However, previous research exploring the time course for HA phenotypes have used fixed load protocols in untrained participants (Tyler & Notley, 2024), with the present study using a mixed‐method protocol in trained individuals, making direct comparisons limited. Still, the demonstrated reductions appear superior to mean changes reported in the updated meta‐analysis by Tyler et al. (2024) for resting core body temperature (−0.16°C) and HR (−5 bpm). The reduction in HR to HA is likely an increase in blood volume due to an increase in PV, possibly from an electrolyte‐mediated enlargement of the extra‐cellular fluid volume (Patterson et al., 2004) and increase in aldosterone. During exercise in hot–humid conditions sweat evaporation is restricted and may be insufficient to compensate the body's metabolic heat production. Therefore, to try to offset the resultant heat strain and cardiovascular challenge, greater PV expansion and retention may be desirable for exercise in hot–humid conditions. In the present study we showed that 7 days of mixed‐method HA significantly increased PV by 9.6%, which is again superior to the mean expansion of 4% reported by Tyler et al. (2024). This has important implications for HA prescription, especially for exercise in hot–humid conditions whereby PV expansion seemingly needs to be optimised compared to sweat loss and rate. In the current study a significant increase in sweat rate was evident after 7 (+23 ± 18%), but not 4 days (+4 ± 21%) of mixed‐method HA. Changes in sweat rate typically follow a slower time course of adaptation (Tyler & Notley, 2024) with longer protocols having a larger effect (STHA: +5 ± 11%; MTHA: +29 ± 29%).

In the current study, participants felt more thermally comfortable (peak TC; −2 AU) and reported a lower perceived exertion (peak RPE; −2 AU) during the cycling HST after four HA exposures, with no further change with an additional three sessions (days 5 vs. 8) in any of the perceptual measures. Unexpectedly, there was no change in mean or peak TSS during the cycling HST after 7 days of HA, despite research by Neal et al. (2016) and Moss et al. (2020) supporting a small effect on reducing mean TSS by 0.9 AU. Nevertheless, these findings demonstrate that 4 days of mixed‐method HA offers a rapid method of heat adaptation which could be used by triathletes travelling to and from hot environments, who in the intervening periods may not be able to maintain adequate heat adaptation (i.e. acclimatisation) specific to the thermal stress of training and competition (Hargreaves, 2008).

The application of mixed‐method HA is currently limited to a case study with a football referee (Ruddock et al., 2016), one study in para‐ and able‐bodied triathletes (Stephenson et al., 2019), another study in elite male rugby sevens athletes (Fenemor et al., 2022) and one study in six international triathletes (James et al., 2024). In the study by Stephenson et al. (2019), 13 triathletes undertook 8 days of consecutive HA: five HR‐controlled training sessions and three passive heat exposures (seated rest in a heat chamber), with an increase in sweat loss (+0.17 L h^−1^) and PV (+6.2%) but no change in resting *T*_re_ (*P* > 0.05). In the study by James et al. (2024), six elite triathletes (three male, three female) undertook eight HA sessions over a 13‐day period: five active sessions within an environmental chamber involving running/cycling‐based exercise to achieve *T*_re_ > 38.5°C and three post‐exercise HWI sessions, with a drop in exercising HR (−6 bpm) after seven HA sessions but no change in core body temperature or PV. Together these previous investigations indicate a mixed active and passive HA protocol can be effective at stimulating thermoregulatory adaptations in officials, endurance trained and elite team sport individuals without modifying training load and having detrimental effects on athlete health or training stress markers. However, in the study by Stephenson et al. (2019) and Fenemor et al. (2022), participants completed HR controlled HA sessions, which may have introduced confounding influences in the HA stimulus due to intra‐individual differences between participants, causing potential complications in matching the adaptation impulse across individuals or when comparing the resulting adaptation between two independent groups (Taylor et al., 2020).

Despite pragmatic reasons for the application of the controlled‐cardiovascular strain technique, the reduced physiological strain across HA sessions may result in a sub‐optimal stimulus for heat adaptation to progress. This is especially important in endurance‐trained individuals who appear to have a lower adaptive response to HA (Taylor, 2014). Controlled hyperthermia protocols provide an elevated thermal impulse which exceeds the individual's threshold for adaptation, ensuring progressive adaptation, and reducing inter‐individual variation in adaptive responses (Taylor, 2014). Another effective method of HA which provides greater flexibility and effectiveness compared to exercise‐based protocols is post‐exercise HWI (McIntyre et al., 2021). This strategy appears to maximise the heat adaptation response via high core and skin temperatures (dual stimulus) during immersion. It seems decisions on which approach is ‘optimal’ may be dependent on cost and athlete preferences, though results in the current study support the use of controlled hyperthermia training combined with post‐exercise HWI as an effective HA strategy for moderately trained triathletes to employ prior to competition in hot, high RH environments.

In the current study no change in 20‐km cycling TT performance in the heat was observed after HA. This was unexpected as medium‐term (8–14 days) HA interventions have been associated with a median improvement of 4% in TT performance (Tyler et al., 2016). During cycling in hot (>30°C) compared with cool conditions (<21°C), a significant increase in core temperature and/or greater TSS is associated with an anticipatory reduction in PO and exercise performance (Peiffer & Abbiss, 2011). Despite a lower mean TSS (−0.6 AU) and end‐exercise *T*_re_ (−0.45°C) in the current study, this did not confer an increase or better maintenance in PO after HA. The 20‐km TT in the heat took on average 34 min in the present study, which may have been too short to allow thermoregulatory‐mediated rises in cardiovascular strain to improve performance in the heat, with PO in the heat shown to be depressed from 20 min onward due to increased skin temperature, increased skin blood flow and reductions in cardiac output (Racinais et al., 2015). Therefore, the benefits of enhanced PV and higher total body water from HA may only come in for performance tests greater than 1 h. Still, these data add to limited evidence on the effects of medium‐term HA on cycling TT performance in hot environments with high RH, which presents a different thermoregulatory challenge compared to exercise in hot temperatures and low RH.

It is important to note that while copeptin and normetanephrine were included as secondary outcomes a priori, their role in this study was exploratory in nature, aimed at evaluating their potential application as adjunct markers of adaptation in endurance athletes. In the current study a reduced sympathetic nervous system excitability with NMET concentrations −24.3% after 7 days of HA was evident. This is in line with several studies that have also reported reduced sympathetic tone during exercise in the heat following HA (Febbraio et al., 1994; Hodge et al., 2013; Nielsen et al., 1993, 1997). For example, research by Hodge et al. (2013) reported a reduction in sympathetic nervous activity (plasma noradrenaline) by 43.7% after 8 days of HA (90‐min walking at 40% ${\dot{V}}_{O_{2}\max}$). Results in the present study are greater than those reported in Stacey, Delves et al. (2018) after 9‐days of natural acclimatisation (−16.4%), most likely due to differences in heat induction used, with controlled hyperthermia in the current study versus a fixed load protocol by Stacey, Delves et al. (2018). Changes in noradrenaline concentration have been shown to be highly correlated with end‐exercise HR (Hodge et al., 2013). As such, it is plausible that noradrenaline's surrogate marker (NMET) would also be highly correlated with changes in peak HR, providing a sensitive marker of HA status alongside HR, helping to inform athlete readiness for competition in the heat.

In the present study there was an attenuated increase in mean copeptin concentration at the post‐HST time point (−26.1%) after 7 days of HA. This is in contrast to the study by Stacey, Woods et al. (2018) who reported no change in copeptin after 9 days of natural heat acclimatisation (−5.6%), despite significant reductions in resting core temperature, sweat sodium concentration and HR. Similar research by Stacey, Delves et al. (2018) suggested that changes in copeptin reflect core body temperature responses more closely than sympathoadrenal markers or osmolality, offering a robust and practical marker of thermal strain. A drop in copeptin in the current study can therefore be associated with the observed drop in peak *T*_re_, HR, and thermal comfort. Fluid intake during HSTs was permitted in the current study compared to refrainment in the study by Stacey, Woods et al. (2018) which may have nullified osmotic and volume stimuli to copeptin release (Thompson et al., 1987). Copeptin concentration reflects water regulation and tonicity, offering a practical surrogate of AVP, with studies on AVP tending to report a decline in levels at the end of exercise following HA (Garrett et al., 2012, 2014; Greenleaf et al., 1981). Reduced copeptin concentration during exercise in the heat may also reflect a reduced risk of tubular injury, as elevations can cause an increase in urine osmolality and reduction in urinary flow, which can lead to an inflammatory kidney state (Mansour et al., 2019). The primary outcomes of this study were changes in physiological markers of heat adaptation (core temperature, heart rate, plasma volume change) and exercise performance during self‐paced running in the heat. Secondary outcomes, specified a priori, included changes in fluid‐regulatory and sympathetic biomarkers (copeptin and normetanephrine) to explore their potential role as practical indicators of heat adaptation status. The inclusion of these biomarkers was based on emerging evidence of their sensitivity to thermal strain and their relevance to health monitoring in both athletic and occupational heat stress contexts.

A key limitation of this study is the selection of biomarkers used to assess physiological strain and heat adaptation. While copeptin and normetanephrine were included as practical, stable indicators of fluid‐regulatory stress and sympathetic nervous system activity, these markers do not provide a complete picture of potential renal stress or AKI risk. The inclusion of more established renal injury biomarkers, such as neutrophil gelatinase‐associated lipocalin (NGAL), would have strengthened the interpretation of heat‐induced strain on kidney function. Future research should incorporate such markers to clarify the relationship between heat adaptation strategies and athlete health, particularly in the context of high training loads and environmental stress.

### 4.1 Conclusions

Combining controlled hyperthermia with post‐exercise HWI offers competitive triathletes an effective and time efficient approach to heat training, inducing significant physiological adaptations with as little as 4 days. An additional 3 days (7 days) of mixed‐method HA had no further benefit on heat adaptation, and despite reductions in thermal strain, had no impact on self‐paced performance in heat compared to a control group. Exploratory findings on copeptin and NMET support a reduction in thermo‐physiological strain after 7 days of mixed‐method HA. These biomarkers have shown potential utility as indirect indicators of both heat adaptation and renal stress, with further research required to validate their utility in large cohorts, ideally alongside more established renal injury markers.

## AUTHOR CONTRIBUTIONS

Daniel Snape conceived the study, collected results, performed data analysis, and drafted the manuscript. Iain T. Parsons collected results and reviewed and revised the manuscript. Michael J. Stacey and David R. Woods critically edited the manuscript and contributed to the content. Barney Wainwright and John O'Hara contributed to the concept and design, review and revised the manuscript, and contributed to data interpretation. All authors have read and approved the final version of this manuscript and agree to be accountable for all aspects of the work in ensuring that questions related to the accuracy or integrity of any part of the work are appropriately investigated and resolved. All persons designated as authors qualify for authorship, and all those who qualify for authorship are listed.

## CONFLICT OF INTEREST

None declared.

## Data Availability

The dataset analysed during the current study may be requested upon an informal inquiry addressed to the corresponding author.
